# Treatment of Dentinal Hypersensitivity by means of Nd:YAP Laser: A Preliminary *In Vitro* Study

**DOI:** 10.1155/2014/323604

**Published:** 2014-10-14

**Authors:** Amaury Namour, Samir Nammour, André Peremans, Daniel Heysselaer, Roeland J. G. De Moor

**Affiliations:** ^1^Department of Operative Dentistry and Endodontology, Ghent Dental Laser Center, Dental School, Ghent University, 9000 Ghent, Belgium; ^2^Department of Dental Sciences, Faculty of Medicine, University of Liege, 4000 Liege, Belgium; ^3^L.A.S.M.O.S, Facultés Universitaires Notre Dame de la Paix, 5000 Namur, Belgium

## Abstract

*Objective*. The aim of this study is to evaluate the effectiveness of Nd:YAP laser to seal dentinal tubules at different parameters. *Material and Methods*. 24 caries-free human wisdom impacted molars were used. The crowns were sectioned transversally in order to totally expose the dentin. The smear layer was removed by a 1 min application of EDTA. Each surface was divided into four quadrants, but only three quadrants were irradiated at a different output power setting (irradiation speed: 1 mm/sec; optical fiber diameter: 320 *µ*m; tangential incidence of beam and in noncontact mode). Samples were smeared with a graphite paste prior to laser irradiation. All specimens were sent for SEM analysis. Pulp temperature increases in additional twenty teeth were measured by a thermocouple. *Results*. Morphological changes in dentin surfaces depend on the value of used energy density. Higher energy densities (2 W–4 W; 200–400 mJ; pulse duration: 100 m sec.; and 10 Hz) induce higher dentin modifications. Our results confirmed that Nd:YAP laser irradiations can lead to total or partial occlusion of dentin tubules without provoking fissures or cracks. Measurements of pulp temperature increases showed that Nd:YAP laser beam can be considered as harmless for pulp vitality for following irradiation conditions: 2 W (200 mJ) to 4 W (400 mJ) with an irradiation speed of 1 mm/sec; fiber diameter: 320 micrometers; 10 Hz; pulse duration: 100 m sec; noncontact mode and in tangential incidence to exposed dentin. The perpendicular incidence of the laser beam on exposed dentin may injure pulp vitality even at low output power of 3 W. *Conclusions*. Nd:YAP laser beam was able to seal the dentin tubules without damaging dentinal surfaces and without harming pulp vitality. Nd:YAP laser is effective and may be safely used for future *in vivo* treatments of dentinal hypersensitivity under certain conditions.

## 1. Introduction

Dentinal hypersensitivity (DH) is described in the literature as a “pain derived from exposed dentin in response to chemical, thermal tactile, or osmotic stimuli which cannot be explained as arising from any other dental defect or disease” [1].

Dentinal hypersensitivity is a quite common problem. Que et al. [2] pointed out a prevalence of dentinal hypersensitivity varying between 2–8% and 74%. Many solutions have been proposed and tested to treat DH but few of them are really successful [3]. DH is a very annoying disease which can have a negative influence on the quality of life, oral hygiene, and treatments like cleanings with ultrasonic instruments.

The etiology of this disease remains unknown, but the most common accepted theory is the fluid movements/hydrodynamic theory proposed by Braennstrom and Astroem, which involves the fluids movements of the tubules. These movements of the fluids are direct reactions of thermal, chemical, osmotic, and mechanical stimuli [4]. The odontoblastic processes are indeed rounded by dentinal fluid coming from the pulp complex, which forms 22% of the dentinal volume [5], and some studies reported that sensitive dentine contain 8 times more tubules, but also wider tubules, than not sensitive teeth [5–7].

It is considered that an ideal desensitizing agent for dentin hypersensitivity should not irritate or endanger the pulp; it should be relatively painless, easily applied, rapid, and permanently effective, and it should not discolor the teeth [8, 9].

The desensitizing methods in use inhibit the pain by trying to avoid any fluid movement or by having an influence on the nerve [10]: sealing dentinal tubules with a coating mechanism that can alter the tubule contents by coagulation, by protein precipitation, or by the creation of insoluble calcium complexes.

Only potassium salts (potassium nitrate) and possibly lasers can have a direct influence on the nerve excitability by disturbing the nerve transmission [11, 12].

Concerning the use of laser for the treatment of laser hypersensitivity, Sgolastra et al. [13] reported that the mechanisms of action of the laser allowing efficient treatment of DH are

1. coagulation of proteins of the fluid inside the dentinal tubules; this will diminish the fluids movements;
2. occlusion of tubules through partial submelting of the denuded dentine;
3. discharging of internal tubular nerve.

Those interesting effects may be acceptable for the clinical use of laser for DH treatment if it is used safely without pulp damage [13, 14].

The aim of our study is to evaluate the ability of the Nd:YAP laser (1340 nm) to induce dentinal melting, to provoke the occlusion of the dentinal tubules, and to determine the safe irradiation conditions.

## 2. Material and Methods

### 2.1. SEM Study

Teeth used in this* in vitro* study were extracted caries-free adult human impacted molars wisdom teeth. Patient age range is 18 to 25 years. Reasons for the extractions were not related to the purpose of this study. 44 caries-free adult human molars were kept in a balanced salt solution at 4°C. 24 teeth were used for the SEM study and 20 were used for the temperature increase study. The external surfaces were cleaned using a scaler, and then crowns were immediately sectioned transversally at low speed (300 rpm) using a precision sectioning 20 LC diamond blade (Isomet Low Speed Saw, Buehler Ltd., Lake Bluff, IL, USA) in order to totally expose the dentin. The anatomical crown and apical part of each root were separated. 3 mm thick dentin discs will be obtained through this procedure. The exposed dentinal surfaces of these discs were polished with Soft-Lex discs 3 M Espe (coarse-grit disc and medium-grit) using a handpiece speed of 12000 rpm for 20 seconds. Then, samples were rinsed with cool water and dried with a five-second air blast.

Each surface was divided into four quadrants with a standard grit diamond bur (C 4, 10 mm long, standard grit, Crosstech Diamond Instruments Ltd., Thailand) under cooling water.

The smear layer was removed by a one-minute application of 18% ethylene diamine tetra-acetic acid (EDTA) (Ultradent Products Inc., USA). Teeth were rinsed with distilled water and immediately irradiated at different energy densities.

The exposed dentins were irradiated with Nd:YAP laser (LOKKI, Lobel Medical, Les Roches de Condrieu, France) as follows: pulsed mode, fiber diameter: 320 *μ*m, tangential incidence of the beam, and in noncontact mode (the distance between the optical fiber and the irradiated surface was 1 to 2 mm). Delivered output power was ranged from 0.9 W to 10 W. The output powers available are predetermined by the manufacturer, so there are only 9 different output powers available on the apparatus (LOKKI laser model): 0.9 W–5 Hz and 0.2 m sec per pulse; 1.4 W–5 Hz and 0.2 m sec; 1.8 W–5 Hz and 0.2 m sec; 2 W–10 Hz and 0.1 m sec; 3 W–10 Hz and 0.1 m sec; 4 W–10 Hz and 0.1 m sec; 5 W–30 Hz and 0.33 m sec; 7.5 W–30 Hz and 0.33 m sec; and 10 W–30 Hz and 0.33 m sec per pulse. Eight teeth were used for each power density. We used a large choice of irradiation parameters for the Nd:YAP laser because of the absence of information in the literature about this kind of laser wavelength. The specimens were placed on a flat surface, the optical fiber was moved by the operator tangentially at approximately 1 mm/sec speed, and the speed was controlled and appreciated by the operator with possible human error. On each tooth, we only irradiated 3 different quadrants. The fourth quadrant was kept as a control without any laser irradiation; it was only treated with EDTA 18% for one minute.

Before laser irradiation, exposed dentine of three quadrants was smeared with a graphite paste prepared by mixing distilled water and fine grain (particle size: 5–25 *μ*m) graphite powder (Pressol, Nuremberg, Germany) as an enhancer. The particle size is larger than the diameter average of dentinal tubules. At the end of the irradiations, samples were carefully rinsed with distilled water in order to eliminate the residual graphite.

An SEM (JSM 7500F, JEOL, Tokyo Japan) study was done in order to find the optimal irradiation parameters of the Nd:YAP laser. The selection criteria were its ability to induce dentinal melting and/or the sealing of tubules without inducing cracks or morphological dentinal destruction. After the metallization of all samples, we used a constant magnification of ×3000 for all SEM examinations.

### 2.2. Temperature Increase Study

We used 20 teeth for the pulp temperature increase measurements. For this part of the study, we decided to only test the optimal irradiation parameters resulting from the SEM analyses that were able to occlude the majority of dentinal tubules.

The cement surfaces of teeth were cleaned using a scaler, and then the cement layer was removed gently with a diamond bur (approximately 100 *μ*m) (C 4, 10 mm long, standard grit, Crosstech Diamond Instruments Ltd., Thailand) in order to totally expose the dentinal tubules. Crowns were sectioned transversally at low speed (300 rpm) using a precision sectioning 20 LC diamond blade (Isomet Low Speed Saw, Buehler Ltd., Lake Bluff, IL, USA) in order to totally expose and open the cameral pulp chamber below the level of the enamel cement margin. The pulp tissue was removed, and the access cavity was cleaned and filled by a special thermoconductor paste having the same thermal conductivity as human tissue: 0.4 cal s^−1^ m^−1^ K^−1^. This is comparable to the thermal conductivity of soft tissues (0.2–0.5 cal s^−1^ m^−1^ K^−1^), depending on hydration [15]. Each tooth was placed into a warm bath at constant temperature of 37°C. A sensor of the K-type thermocouple (K-type thermocouples HH806AWE Omega, Manchester, UK) was placed into the pulp chamber against the dentinal wall in regard to the future irradiated zone by occlusal access. The second sensor of the thermocouple was placed into the warm bath in order to control the constancy of water temperature at 37°C. We started each measurement after verification that intrapulpal temperature was stable at 37°C.

The graphite paste was applied on external dentinal surfaces below the cervical border (enamel/dentine junction) on a surface of *h*: 2 mm ×*L*: 5 mm.

The treatment of each area covered by the graphite was performed with the Nd:YAP laser beam in tangential incidence with an approximate speed of 1 mm/sec. Between two successive temperature measurements, we also took care to wait enough time in order to allow irradiated dentin to have a thermal relaxation and to allow the pulp chamber to once again stabilize its temperature at 37°C.

We performed 6 measurements per irradiation parameter.

According to the study of Zach and Cohen [16], we considered the temperature increase as safe when it was below the trigger temperature of 3°C.

## 3. Results

### 3.1. SEM Analysis

**Figure 1:**
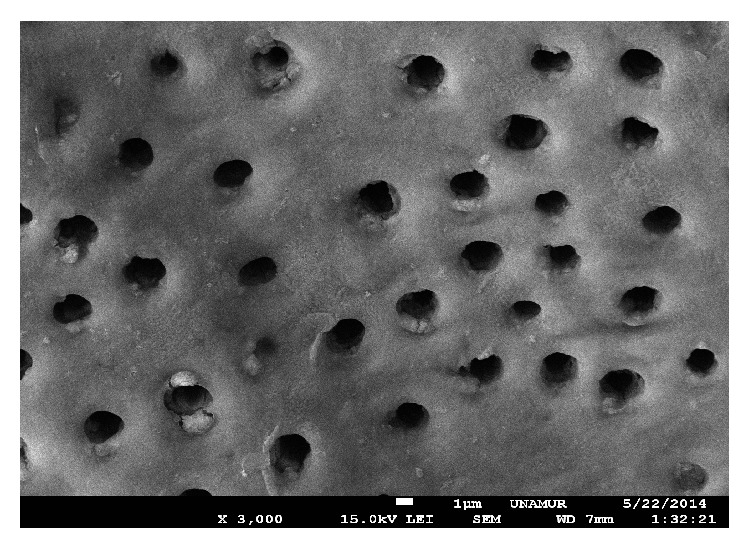
SEM view of unlased dentin (control) treated only with EDTA (18%) during one minute. The dentin is not covered by the smear layer. The tubules are open. Magnification: 3000x.

The unlased dentin of the control groups which were only treated with EDTA showed a dentinal surface without the smear layer and wide open tubules (Figure 1).

Dentinal surfaces irradiated by means of Nd:YAP laser beam showed different structural changes depending on the delivered power. We observed a direct correlation between the power settings and the tubules occlusion of the exposed dentin.

**Figure 2:**
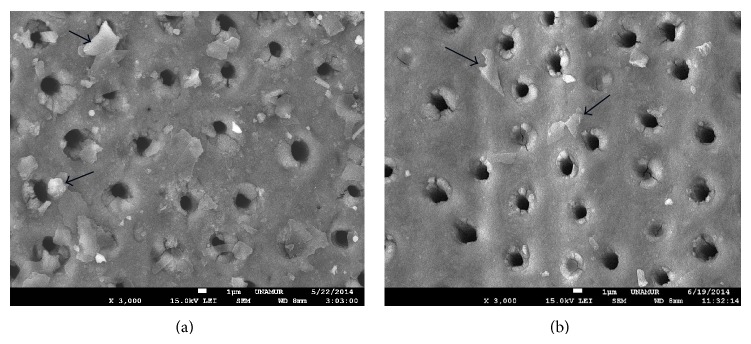
SEM view of Nd:YAP lased dentin treated previously with EDTA (18%). SEM view of exposed dentine with Nd:YAP laser beam at 0.9 W (a) and 1.4 W (b). We can only notice a slight tubules narrowing. Arrows show the graphite particles still existing on the dentinal surface (not disintegrated by the laser beam). Magnification: 3000x.

The output power that ranged from 0.9 W to 1.4 W did not allow occlusion of tubules (Figure 2).

**Figure 3:**
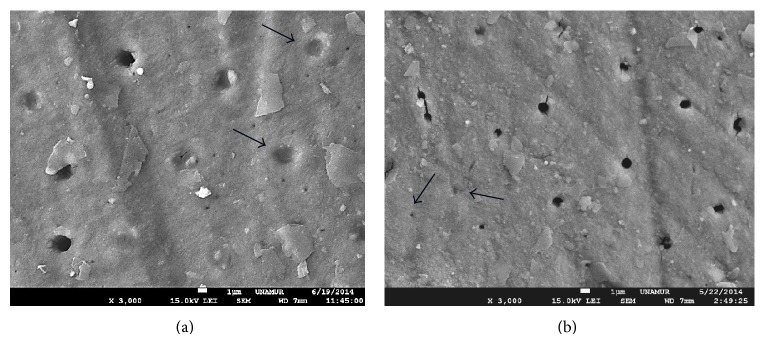
SEM view of Nd:YAP lased dentin treated previously with EDTA (18%). SEM view of exposed dentine with Nd:YAP laser beam at 1.8 W (a) and 2 W (b). We can notice a tubules narrowing. Arrows show some occluded tubules. Magnification: 3000x.

The output power that ranged from 1.8 W to 2 W induced a tubules narrowing and some total tubules occlusion (Figure 3).

**Figure 4:**
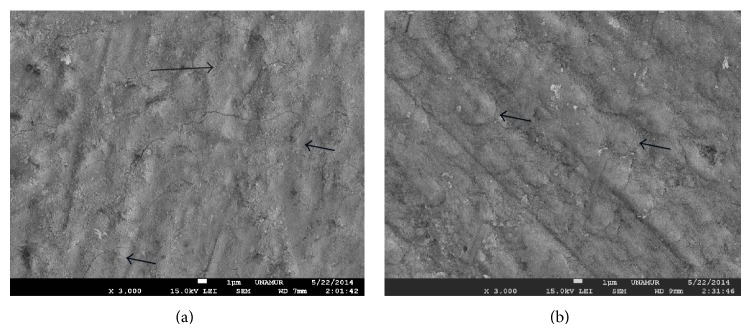
SEM view of Nd:YAP lased dentin treated previously with EDTA (18%). SEM view of exposed dentine with Nd:YAP laser beam at 2 W (a) and 3 W (b). We notice a total occlusion of tubules. Arrows show some total occluded tubules. Magnification: 3000x.

Only output power ranging from 3 W to 4 W can induce a total occlusion of tubules (Figure 4).

**Figure 5:**
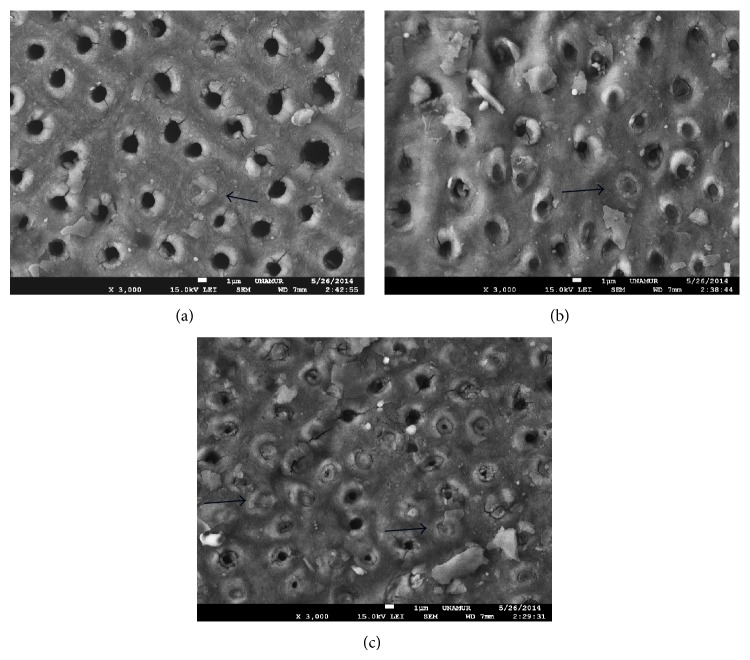
SEM view of Nd:YAP lased dentin treated previously with EDTA (18%). SEM view of exposed dentine with Nd:YAP laser beam at 5 W (a), 7.5 W (b), and 10 W (c). We can notice a tubules narrowing specifically in (b) and (c). Arrows show some occluded tubules. Magnification: 3000x.

Higher power settings ranging between 5 W and 10 W with reduced pulse duration induced limited total occlusion or tubules narrowing (Figure 5).

### 3.2. Temperature Increase

In this part of the pulp temperature increase, we decided to only test the optimal irradiation parameters resulting from the SEM study that were able to occlude the majority of dentinal tubules: 2 W, 3 W, 4 W, and 5 W in pulsed mode.

**Figure 6:**
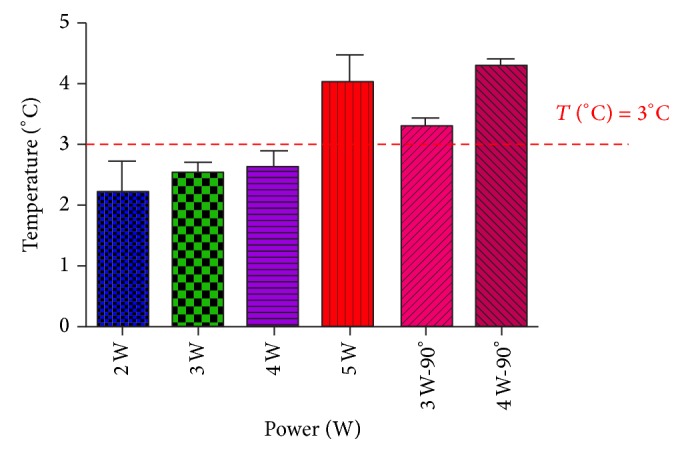
Pulp temperature rise during Nd:YAP laser irradiation of exposed dentine for tubular occlusion. The output powers ranging between 2 W and 4 W used with a tangential incidence may be considered as harmless for the pulpal vitality.

The output powers ranging between 2 W and 4 W used with a tangential incidence of the laser beam induced a pulpal temperature increase lower than the trigger point of 3°C, while higher power settings induced temperature increases above 3°C (Figure 6). It is interesting to note that output parameters considered as harmless (3 W and 4 W) generated pulpal temperature increase higher than 3°C when the incidence of the laser beam is used perpendicularly to the dentinal surfaces (Figure 6).

All values passed the normality test (Kolmogorov-Smirnov test with Dallal-Wilkinson-Lillie for *P* value).  Table 1 shows the means and standard deviations of pulpal temperature rise for each irradiation condition.

## 4. Discussion

We selected young wisdom teeth in our study with the aim of obtaining samples that were as homogenous as possible with similar degrees of dentinal calcification in order to evaluate the effectiveness of Nd:YAP laser to melt dentine and to close wide open tubules.

Ethylene diamine tetra acetic acid (EDTA) is a chelating agent of calcium ions that induces the demineralization of the dentine and the smear layer removal [17]. We decided to treat all exposed dentinal surfaces of samples with EDTA in order to have tubules totally open [18]. In this way, we wanted to simulate the same clinical situation of open tubules causing the dentinal hypersensitivity.

The physical properties of each laser wavelength influence the level of absorption and interaction with each tissue. The Nd:YAP laser wavelength is not well absorbed by hard dental tissue to be able to heat sufficiently the surface of the dentine without inducing a pulp overheating. For this reason, we decided to use the graphite paste applied on the surface of the dentine in order to use a lower output power of the laser than would be necessary without the graphite paste [19]. The absorption of Nd:YAP laser beam by the graphite generates a sudden increase in temperature that would be able to provoke an immediate superficial dentinal melting leading to partial occlusion or narrowing of dentinal tubules. We also used pulsed modes in order to allow dentin to have a thermal relaxation. We selected the noncontact mode to avoid the damage of optical fiber from the heating of the graphite paste.

Our laser beam struck the dentinal surface at a tangential angle, with the aim of avoiding a direct pulp exposure by the nonabsorbed part of the beam by dentin. We selected the optimal irradiation conditions generating a pulpal temperature increase below 3°C [16]. The tangential mode is indicated because the reduction of the incident angle towards the refractive angle of the tissue surface increases the potential for true light reflection with an important reduction of pulp absorption of incident beam. Our results showed that this precaution was justified. In fact, some harmless parameters (3 W and 4 W used with a tangential incidence) showed a dramatic increase in pulp temperature (higher than 3°C) when they were applied in perpendicular incidences. However, it remains a potential bias for recording the elevation of the pulp temperature. Furthermore, in case of using a perpendicular incidence, we may provoke an artificial pulp temperature increase because the 1430 nm wavelength is well absorb by the metal and can induce possible electromagnetic interferences with the metallic sensor of the thermocouple.

In previous studies, authors demonstrated the possibility to occlude dentinal tubules by means of different wavelengths. Kim et al. [20] demonstrated the feasibility of tubules occlusion by using a CO_2_ laser and nanocarbonate apatite, while Han et al. [21] also succeeded in occluding dentinal tubules by replacing the CO_2_ laser by an Er:YAG laser.

Umana et al. [19] succeeded in occluding dentinal tubules using diode lasers (810 nm and 980 nm) in combination with graphite paste. Farmakis et al. [22] showed the possibility to occlude tubules using the combination of bioglass and Nd:YAG laser.

Further studies should be conducted in order to evaluate the clinical efficiency of Nd:YAP laser for dentinal hypersensitivity treatment. It is also necessary to evaluate the clinical persistence of this treatment using Nd:YAP and graphite paste.

## 5. Conclusions

Under the limitations of this study, the combination of an Nd:YAP laser and a graphite paste is able to induce tubule occlusion, and it may be recommended for a future safe clinical application. Our results pointed out that the following parameters can be considered as efficient for tubules occlusion and harmless for dental pulp: 2 W (200 mJ) to 4 W (400 mJ) with an irradiation speed of 1 mm/sec; fiber diameter: 320 micrometers; 10 Hz; pulse duration: 100 m sec; nonfiber contact mode and in tangential incidence to exposed dentin. The perpendicular incidence of the laser beam on exposed dentin may injure pulp vitality even at low output power of 3 W.

## Figures and Tables

**Table 1:** Means and standard deviations for each irradiation condition. All groups passed the normality test of Kolmogorov-Smirnov test (with Dallal-Wilkinson-Lillie for *P* value).

	2 W	3 W	4 W	5 W	3 W—90°	4 W—90°
Number of values	6	6	6	8	8	8

Mean	2,180	2,525	2,620	4,038	3,300	4,300
Std. deviation	0,5450	0,3500	0,2864	1,269	0,1414	0,1000
Std. error	0,2437	0,1750	0,1281	0,4488	0,1000	0,05774

KS normality test						
KS distance	0,1871	0,1723	0,2722	0,1951	0,1788	0,1812
*P* value	>0.10	>0.10	>0.10	>0.10	>0.10	>0.10
Passed normality test (alpha = 0.05)?	Yes	Yes	Yes	Yes	Yes	Yes
*P* value summary	ns	ns	ns	ns	ns	ns
